# ‘Latent’ Portal Hypertension in Benign Biliary Obstruction

**DOI:** 10.1155/1996/21750

**Published:** 1996

**Authors:** Md. Ibrarullah, S. S. Sikora, D. K. Agarwal, V. K. Kapoor, S. P. Kaushik

**Affiliations:** 1 Department of Surgical Gastroenterology Sanjay Gandhi Postgraduate Institute of Medical Sciences Lucknow 226014 India; 2 Department of Gastroenterology Sanjay Gandhi Postgraduate Institute of Medical Sciences Lucknow 226014 India

## Abstract

A prospective study was undertaken to evaluate the changes in portal venous pressure in patients with benign biliary obstruction (BBO) but without overt clinical, endoscopic or radiological evidence of portal hypertension. Portal venous pressure was measured at laparotomy in 20 patients (10 each with either benign biliary stricture or choledocholithiasis) before and after biliary decompression. Pressure was found to be on the high side in seven patients (>25 cm of saline in three patients and > 30 cm of saline in four). The mean fall of pressure was 3.4 cm of saline after biliary decompression. No correlation could, however, be found between portal venous pressure and duration of biliary obstruction, serum bilirubin or bile duct pressure. Liver histology showed mild to moderate cholestatic changes but maintained portal architecture in all. Benign biliary obstruction may therefore, lead to elevation of portal pressure, even though the patient may not necessarily have any clinical, endoscopic or radiological manifestations of portal hypertension. The pathogenesis of this ‘latent’ portal hypertension is probably multifactorial. If biliary obstruction is left untreated the development of overt portal hypertension may become a possibility in the future.


*HPB Surgery*, 1996, Vol. 9, pp.149–152
Reprints available directly from the publisher
Photocopying permitted by license only

© 1996 OPA (Overseas Publishers Association)
Amsterdam B.V. Published in The Netherlands
by Harwood Academic Publishers GmbH
Printed in Malaysia

### 'Latent' Portal Hypertension in Benign Biliary Obstruction

MD. IBRARULLAH, S. S. SIKORA, D. K. AGARWAL,* V. K. KAPOOR and S. P. KAUSHIK

Departments of Surgical Gastroenterology & Gastroenterology*,
Sanjay Gandhi Postgraduate Institute of Medical Sciences, Lucknow 226014, India

A prospective study was undertaken to evaluate the changes in portal venous pressure in patients with benign biliary obstruction (BBO) but without overt clinical, endoscopic or radiological evidence of portal hypertension. Portal venous pressure was measured at laparotomy in 20 patients (10 each with either benign biliary stricture or choledocholithiasis) before and after biliary decompression. Pressure was found to be on the high side in seven patients (>25 cm of saline in three patients and > 30 cm of saline in four). The mean fall of pressure was 3.4 cm of saline after biliary decompression. No correlation could, however, be found between portal venous pressure and duration of biliary obstruction, serum bilirubin or bile duct pressure. Liver histology showed mild to moderate cholestatic changes but maintained portal architecture in all. Benign biliary obstruction may therefore, lead to elevation of portal pressure, even though the patient may not necessarily have any clinical, endoscopic or radiological manifestations of portal hypertension. The pathogenesis of this 'latent' portal hypertension is probably multifactorial. If biliary obstruction is left untreated the development of overt portal hypertension may become a possibility in the future.

KEY WORDS: Biliary cirrhosis obstructive jaundice portal hypertension

### INTRODUCTION

Prolonged extrahepatic biliary obstruction is known to cause portal hypertension subsequent to secondary biliary cirrhosis, in humans as well as in experimental animals. Biliary decompressive surgery in these patients is a formidable undertaking and may not necessarily reverse or ameliorate the portal hypertension[1]. The changes in portal venous pressure (PVP) in the early stages of biliary obstruction have not been well documented. The present study was, therefore, undertaken to evaluate on a prospective basis, the changes in portal venous pressure (PVP), if any, in patients with benign biliary obstruction (BBO), who do not have any evidence of clinical, endoscopic or radiological overt portal hypertension and its correlation with the duration of biliary obstruction, serum bilirubin and bile duct pressure.

*Correspondence to*: S.P. Kaushik, Professor & Head, Dept. of Surgical, Gastroenterology, S G P G I M S, Lucknow 226014, India

### PATIENTS AND METHODS

Ten patients with BBO due to postcholecystectomy stricture (Group A) and 10 with choledochlithiasis (Group B) were recruited for this study. All the patients had jaundice at presentation or in the past and dilated intrahepatic biliary radicles on ultrasonography. Presence of overt portal hypertension was ruled out by clinical examination, endoscopy and ultrsasonography. Ultrasonography was also used to further rule out the presence of extrahepatic portal venous obstruction.

The duration of biliary obstruction was considered as the interval between the first appearance of jaundice and admission to hospital for definitive surgery. The biochemical parameters of liver function were recorded. After laparotomy, PVP was recorded before starting any major dissection, by cannulating a moderate size vein in the gastrocolic ligament or the gastroepiploic venous arcade with a 20–22G needle connected to a pressure recorder, taking the heart as the base line. The surgery was carried out as required. Bile duct pressure was recorded by direct puncture of the extrahepatic bile duct before the definitive Surgery. After the surgery, PVP was recorded once again as before. A liver biopsy was taken and the abdomen was closed in layers. Portal venous pressure upto 25 cm of saline was considered normal, 25–30 cm equivocal and >30 cm definitely elevated. The portal pressures were also correlated with the duration of obstruction, levels of serum bilirubin and pressure within the bile ducts.

### *Statistical method*

Correlation was calculated using Pearson's correlation coefficient.

### RESULTS

There were 9 males and 11 females; the mean age was 38 years. The mean duration of biliary obstruction, serum bilirubin, PVP before and after biliary decompression and bile duct pressure before decompression are shown in the table. At surgery no collaterals suggestive of portal hypertension were found in any patient. In four patients it was not possible to record bile duct pressure because of the presence of multiple stones and/or sludge.

**Table** Duration of biliary obstruction, serum bilirubin, portal venous pressure and bile duct pressure in patients of benign biliary obstruction (Group A: biliary stricture, Group B: choledocholithiasis)

| | *Group A mean (range)* | *Group B mean (range)* |
|---|---|---|
| Duration of biliary obstruction in weeks | 17.8 (4–56) | 27.4 (2–208) |
| Serum Bilirubin in mg/dl | 10.4 (3.8–20) | 8.8 (1.3–21) |
| PVP in cm of saline | | |
| before biliary decompression | 20.5 (12–36) | 22.7 (17–38) |
| after biliary decompression | 17.4 (11–32) | 18.9 (16–31) |
| Bile duct pressure in cm of saline | 21.2 (16–32) | 31 (11–47) |

PVP was equivocally elevated i.e. more than 25 cm of saline in three patients (Group A 1, Group B 2) and definitely elevated i.e. more than 30 cm of saline in four patients (Group A 1, Group B 3). The mean fall of PVP was 3.4 cm of saline (range 0–11) after corrective surgery. No correlation was found between PVP and duration of biliary obstruction (fig. 1, r = 0.43, p = NS), serum bilirubin (fig. 2, r = 0.07, p = NS) or bile duct pressure (fig. 3, r = 0.10, p = NS).

Liver histology showed widening of portal tract, bile plugs, bile duct proliferation, a minimal to moderate degree of portal fibrosis and inflammatory cell infiltrates in all patients. Portal architecture was however maintained in all.

### DISCUSSION

Since the portal venous system is a valveless system, cannulation of one of its tributaries i.e. omental vein, mesenteric vein or umbilical vein has been accepted to represent PVP at laparotomy[2,3]. The normal range of PVP recorded at laparotomy is reported to be 8.5–30 cm of saline[2,4,5]. The PVP in patients with overt portal hypertension has ranged between 25–55 cm of saline[2,6]. The wide range may, in part, be due to the variation in resistance interposed between the measuring site in the portal vein tributaries and the portal vein itself[3] and the effect of anaesthesia on splanchnic circulation which may also vary in different patients at different times during surgery[7].

The omental and gastroepiploic vein was used to measure portal pressure in the present study. PVP was found to be elevated in 7/20 (35%) patients (equivocal 3, definite 4) with BBO. This is a significant finding as none of the patients in the present study had clinical, endoscopic or radiological manifestation of portal hypertension. Liver biopsy did not show evidence of secondary biliary cirrhosis in any of these patients. Though such an observation has been made before in experimental animals[8], no such measurements have been done objectively in human beings.

Raised PVP in patients with BBO may be due to compression or obstruction of intrahepatic portal venous radicles, the causes of which may well be regenerating liver nodules in secondary biliary cirrhosis, portal fibrosis, dilated bile ducts, bile ductular proliferation, venous thrombosis and raised intrahepatic hydrostatic pressure[8–13].

The present study does not point towards any dominant factor contributing to PVP elevation. All factors except regenerating nodules may well have contributed to increased PVP. No correlation was found between the PVP and duration of biliary obstruction, bile duct pressure or the level of serum bilirubin. The distended bile ducts contributing to raised PVP by compression of portal venous radicles[10,12] can only be found in the early stages of biliary obstruction when the proliferative changes in the liver histology are

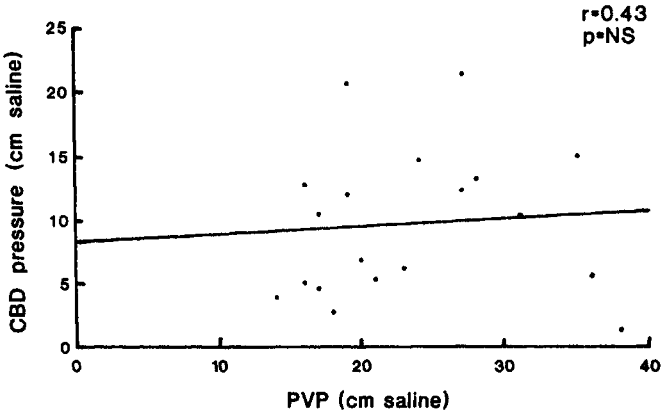

**Fig. 1** Portal venous pressure (PVP) and duration of biliary obstruction, no linear correlation, r = 0.43, p = NS

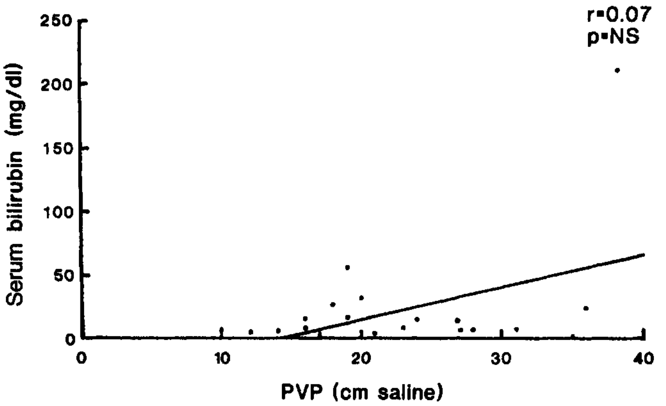

**Fig. 2** PVP and serum bilirubin, no linear correlation, r = 0.07, p = NS

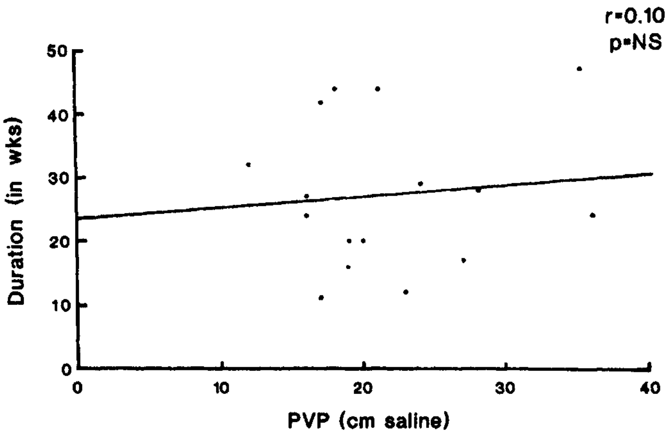

**Fig. 3** PVP and bile duct pressure, no linear correlation, r = 0.10, p = NS

minimal. The intraoperative drop in PVP, as was observed in some patients at the close of surgery could have been due to decompression of the distended bile ducts. The additional possibility of changes in splanchnic flow or resistance, secondary to the effect of anesthesia, however, can not be ruled out.

Portal fibrosis secondary to long standing biliary obstruction has been identified as an important contributor to the rise in PVP in experimental animals as well as in human beings[8,9,13]. But, in the present study no direct correlation could be found between PVP and duration of biliary obstruction. The reasons for this discrepancy could be two; firstly, the duration of biliary obstruction, considered from the day of appearance of jaundice by others[13] as well as in this study could well be fallacious, since the latter only represents complete bile duct obstruction and it is well established that liver changes can occur even with incomplete or intermittent obstruction. Further, the liver fibrosis is also influenced by the episodes of cholangitis accompanying biliary obstruction[1,13]. Therefore in an individual case it is difficult to predict the degree of fibrosis and the possible rise of PVP on the basis of duration alone.

It is interesting to know that none of the patients in the present study despite having PVP in the range of 'established' portal hypertension had associated venous collaterals or gastroesophageal varices. It has been reported that a PVP and inferior venacaval pressure gradient of 12 mm Hg is required for the development of collateral circulation[15] besides some unidentified factors for angiogenesis[16].

The present study could well be criticized on the basis that no effort has been made to record the portal pressure gradient; therefore it is possible that despite recorded high PVP the required gradient may not have been achieved for development of collaterals. It may also be possible that given adequate time (the limit not as yet defined), varices could still develop consequent to the high pressure recorded in these patients, if they were not interfered with and their obstruction surgically corrected.

To conclude, therefore, benign biliary obstruction does seem to lead to elevation of PVP. There may not necessarily be any obvious clinical, endoscopic or radiological manifestations of portal hypertension in these patients. The pathogenesis of this 'latent' portal hypertension is probably multifactorial. If left untreated, these patients have a potential risk of developing overt portal hypertension and its complications later on.

### REFERENCES

1. Benjamin, I.S. (1988). Biliary tract obstruction–pathophysio logy. In: Blumgart L.H. (ed.) Surgery of the liver and biliary tract. 1st ed, pp 111–120. Edinburgh: Churchill Livingston.
2. Taylor, F.W., Egbert, H.L. (1951) Portal tension. *Surg Gynecol Obstet*, **92**, 64–68.
3. Reynolds, T.B., Ito, S., Iwatsuki, S. (1970) Measurements of portal pressure and its clinical application. *Am J Med*, **49**: 649–657.
4. Bellis, C.J. (1942) Portal venous pressure in man. *Proc Soc Exp Biol Med*, **50**, 258–264.
5. Moreno, A.H., Chiesa, D., Affani, J. (1957) Studies on portal tension of human adults. *Surg Gynecol Obstet*, **104**, 25–30.
6. Thompson, W.P., Caughey, J.L., Whipple, A.O., Rousselot, L.M. (1937) Splenic vein pressure in congestive splenomegaly (Banti's syndrome). *J Clin Invest*, **16**, 571–578.
7. Debaene, B., Goldfarb, G., Braillon, A. (1990) Effect of ketamine, halothane, enflurane and isoflurane on systemic and splanchnic hemodynamics in normovolemic and hypovolemic cirrhotic rats. *Anesthesiology*, **73**, 118–124.
8. Franco, D., Gigon, M., Szekely, A.M., Bismuth, H. (1979) Portal hypertension after bile duct obstruction. *Arch Surg*, **114**, 1064–1067.
9. Bosch, J., Enriguez, R., Groszmann, R.J., Storer, E.H. (1983) Chronic bile duct ligation in dog: hemodynamic characterization of a portal hypertensive model. *Hepatology*, **3**, 1002–1007.
10. Beduhn, D., Kampmann, H., Encke, A. (1974) Vergleichende angiographische und szintigraphische un tersuchungen beim experimentellen parenchym und verschlussikterus. *Radiologe*, **13**, 11–14.
11. Rutherford, R.B., Boitnott, J.K., Donohoo, J.S. (1970) The production of biliary cirrhosis in macaca mulatha monkeys. *Arch Surg*, **100**, 55–63
12. Reuter, S.R., Chuang, V.P. (1976) The location of increased resistance to portal blood flow in obstructive jaundice. *Investigative Radiol*, **11**, 54–59.
13. Scobie, B.A., Summerskill, W.H. (1965) Hepatic cirrhosis secondary to obstruciton of the biliary system. *Am J Digest Dis*, **10**, 135–146.
14. Benjamin, I.S. (1982) The obstructed biliary tract. In: Blumgart, L.H. (ed.) The biliary tract. Clinical Surgery International vol 5, pp 157–217. Edinburgh: Churchill Livingston
15. Franchis, R., Primignani, M. Why do varices bleed. *Gastroenterol Clin North Am*, **21**, 85–101.
16. Bosch, J., Pizmeta, P., Feu, F., Fernandez, M., Gracia-Pagan, J.C. (1992) Pathophysiology of portal hypertension. *Gastroenterol Clin North Am*, **24**, 1–14.

